# A unique thermo-induced gel-to-gel transition in a pH-sensitive small-molecule hydrogel

**DOI:** 10.1038/s41598-017-09304-z

**Published:** 2017-08-16

**Authors:** Hongtao Xie, Mehran Asad Ayoubi, Wensheng Lu, Jide Wang, Jianbin Huang, Wei Wang

**Affiliations:** 1 Ministry Key Laboratory of Oil and Gas Fine Chemical, College of Chemistry and Chemical Engineering, Xinjiang University, Urumqi, 830046 China; 2 Novel Drug Delivery Systems Department, Iran Polymer and Petrochemical Institute, P.O. Box 14975/112, Tehran, Iran; 3 Beijing National Laboratory for Molecular Sciences, Key Laboratory of Colloid, Interface and Chemical Thermodynamics, Institute of Chemistry, Chinese Academy of Sciences, Beijing, 100190 China; 4 Beijing National Laboratory for Molecular Sciences (BNLMS), State Key Laboratory for Structural Chemistry of Unstable and Stable Species, College of Chemistry and Molecular Engineering, Peking University, Beijing, 100871 China; 5 Department of Chemistry and Center for Pharmacy, University of Bergen, Bergen, N-5007 Norway

## Abstract

For a hydrogel based on a zwitterionic dendritic surfactant, we report an apparently unprecedented reversible temperature-induced gel-to-gel phase transition below the melting point of its alkyl chains, where the supramolecular self-assembly of surfactant molecules underwent a dramatic transformation from low-temperature surfactant bilayers to high-temperature entangled surfactant worm-like micelles.

## Introduction

Gels undergoing rapid morphological changes in response to external stimuli such as heat^1^, pH or ion change^2^ have become important in the applied material science and the theoretical regime. For responsive gel systems, the phenomenon of thermo-induced gel-to-gel transition is particularly interesting. For example, poly(N-isopropylacrylamide) (PNIPAAm) hydrogels exhibit a volumetric phase transition at their lower critical solution temperatures. This property has led to many promising applications, such as catalysis^3^, drug delivery^4^, protein chromatography^5^, and tissue and cell surface engineering^6^. For low-molecular-weight gelators (LMWGs), conversely, thermo-induced gel-to-gel transition is very rare. Up to now, temperature-induced gel-to-gel transition in LMWG hydrogels is limited to a few cases^7, 8^. Meister *et al*.^8^. showed a temperature-induced gel-to-gel transition for bola amphiphiles when the alkyl chain length exceeded 26 carbon atoms. Both gel phases of the bola amphiphiles were composite of fibers^8^. Kotlewski et al^7^. reported a hard-to-soft organogel transition. The system exhibited a series of transitions from a discotic columnar to a plastic crystal to a crystalline phase. The soft organogel only existed in a very narrow temperature range (<5 °C). The realization of such transitions in LMWG hydrogels is important, because it may open new horizons for applications of such materials in many industrial fields, from sensors^9^ and templating materials^10^ to drug delivery^11^.

The gel-forming capability of a LMWG is based on its ability in constructing a self-assembled fibrillar network (SAFiN) that can trap solvent molecules through actions of capillary forces. Within a SAFiN, the basic supramoelcular packing of LMWG molecules involves, e.g., lamellar^12^, micellar^13^, helical^12, 14^, or crystalline^15^ structures. In the vast majority of cases, increasing the temperature of a LMWG hydrogel causes a gel-to-sol transition^1^. This happens because the balance between gelator-gelator and gelator-solvent interactions that induced formation of a solvent-trapping SAFiN in the first place^16^ is disrupted. This means that for the realization of a thermo-induced gel-to-gel transition, the balance of supramolecular interactions should dictate transformations between distinctly different types of solvent-trapping SAFiNs once the temperature is changed.

**Figure 1:**
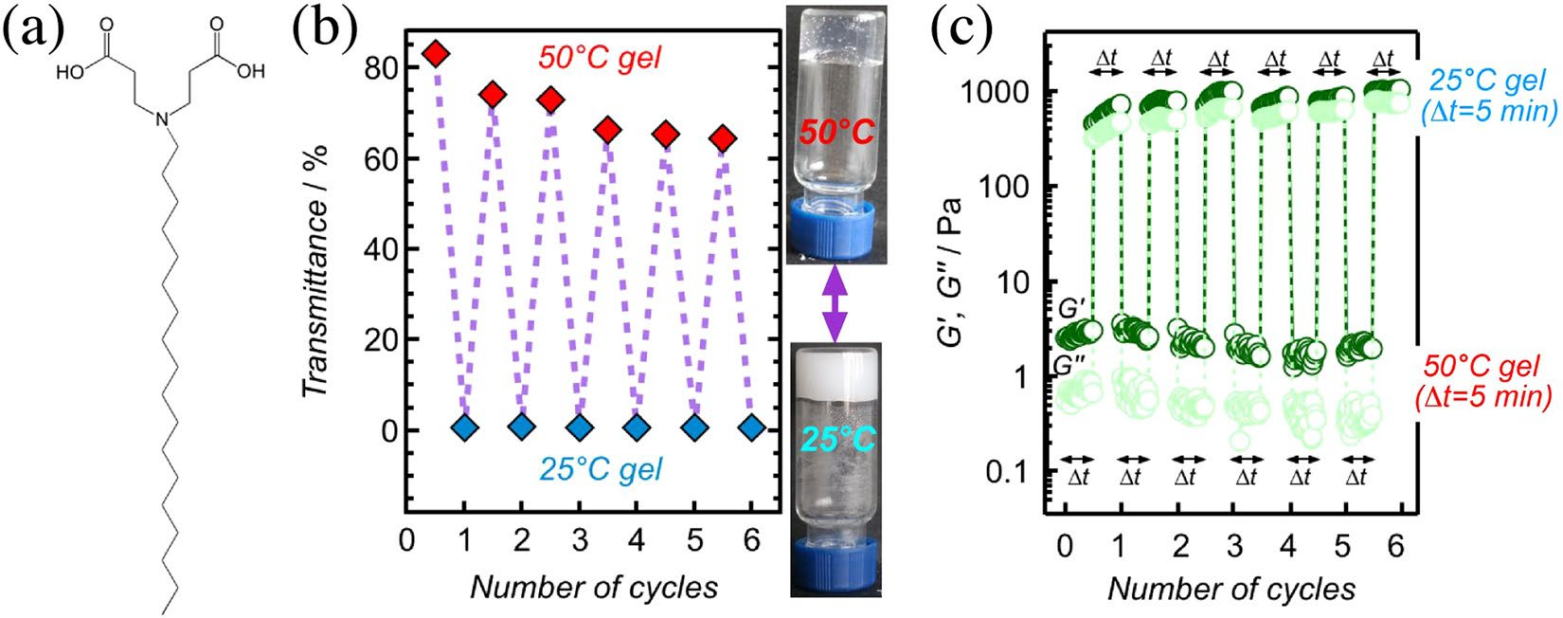
(**a**) Chemical formula of C18ADPA. (**b**) Pictures of 2% pH = 5.37 hydrogel at 25 °C and 50 °C together with its transmittance data during successive cooling-heating cycles. (**c**) Rheological data during successive cooling-heating cycles (strain = 0.5%; angular frequency of ω = 10 rad/s). In (**b**) and (**c**), the waiting time after a temperature increase was about 2 min and after a temperature decrease was about 5 min.

Herein, we show that a hydrogel of zwitterionic amphiphilie 3,3′-(octadecylazanediyl)dipropionic acid (C18ADPA) [Fig. 1a] exhibited an unprecedented reversible temperature-induced *gel-to-gel* phase transition. The transition was unique, because it involved a bilayer-to-micelle transition. More specifically, a low-temperature crystalline branched-tubular gel (studied at 25 °C) changed into a high-temperature entangled worm-like micellar gel (studied at 50 °C). This means that by adjusting the temperature either of these two fundamentally different mechanisms of network formation^17^ could be activated. Also, hydrogels of C18ADPA featured a pH-triggered gel-to-sol transition, a narrow pH-window for their stability, and a pH-dependent viscosity [Supplementary Figs S2–S4; in the rest of the paper we focus on pH = 5.37 hydrogel (2% w/v), for which, the carboxylic acid groups and the tertiary amine were both ionized^18^].

## Results and Discussion

**Figure 2:**
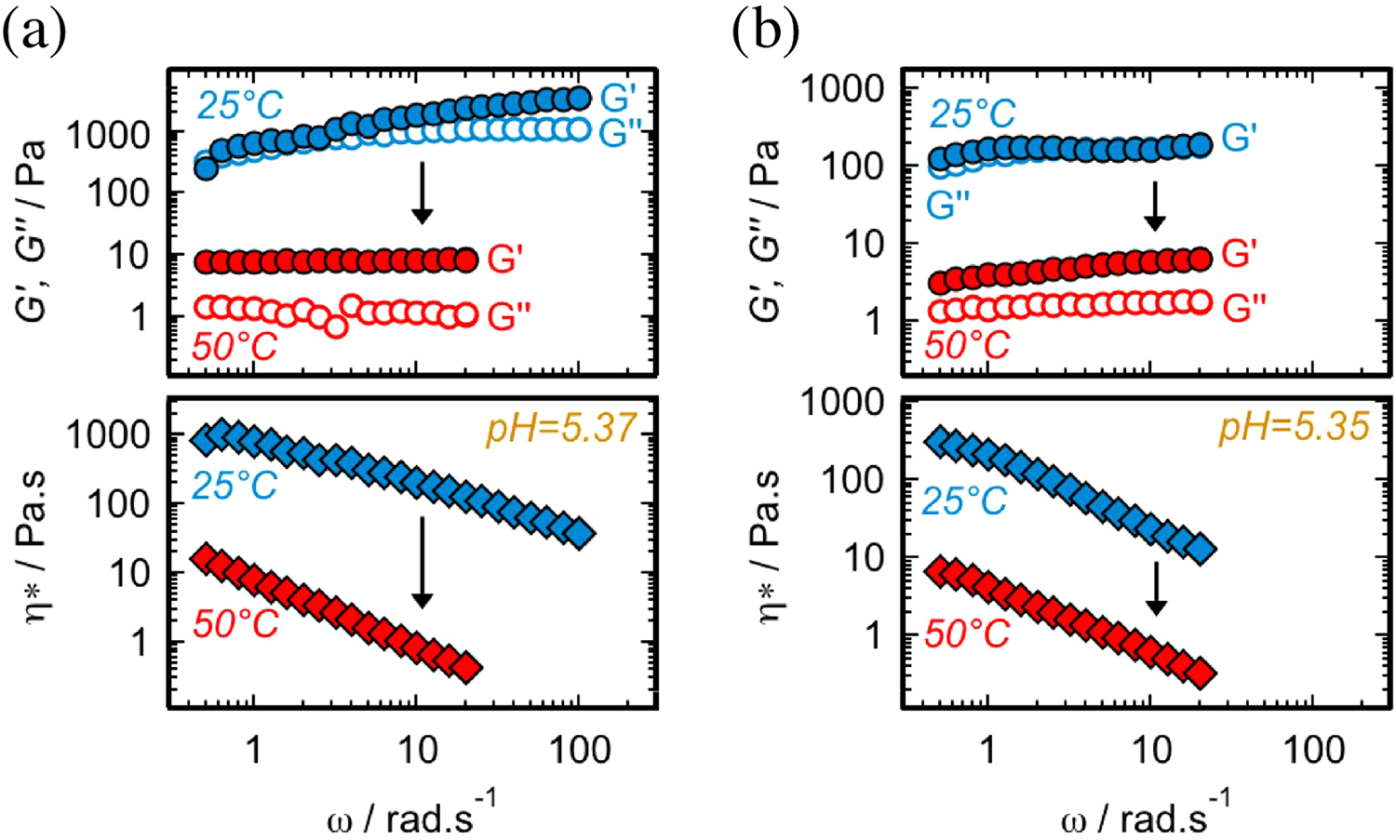
Frequency sweep dynamic rheological data for different hydrogel systems. (**a**) 2% pH = 5.37 hydrogel at 25 °C (strain = 0.3%) and 50 °C (strain = 2%), and (**b**) 2% pH = 5.35 hydrogel at 25 °C and 50 °C (strain = 2%).

**Figure 3:**
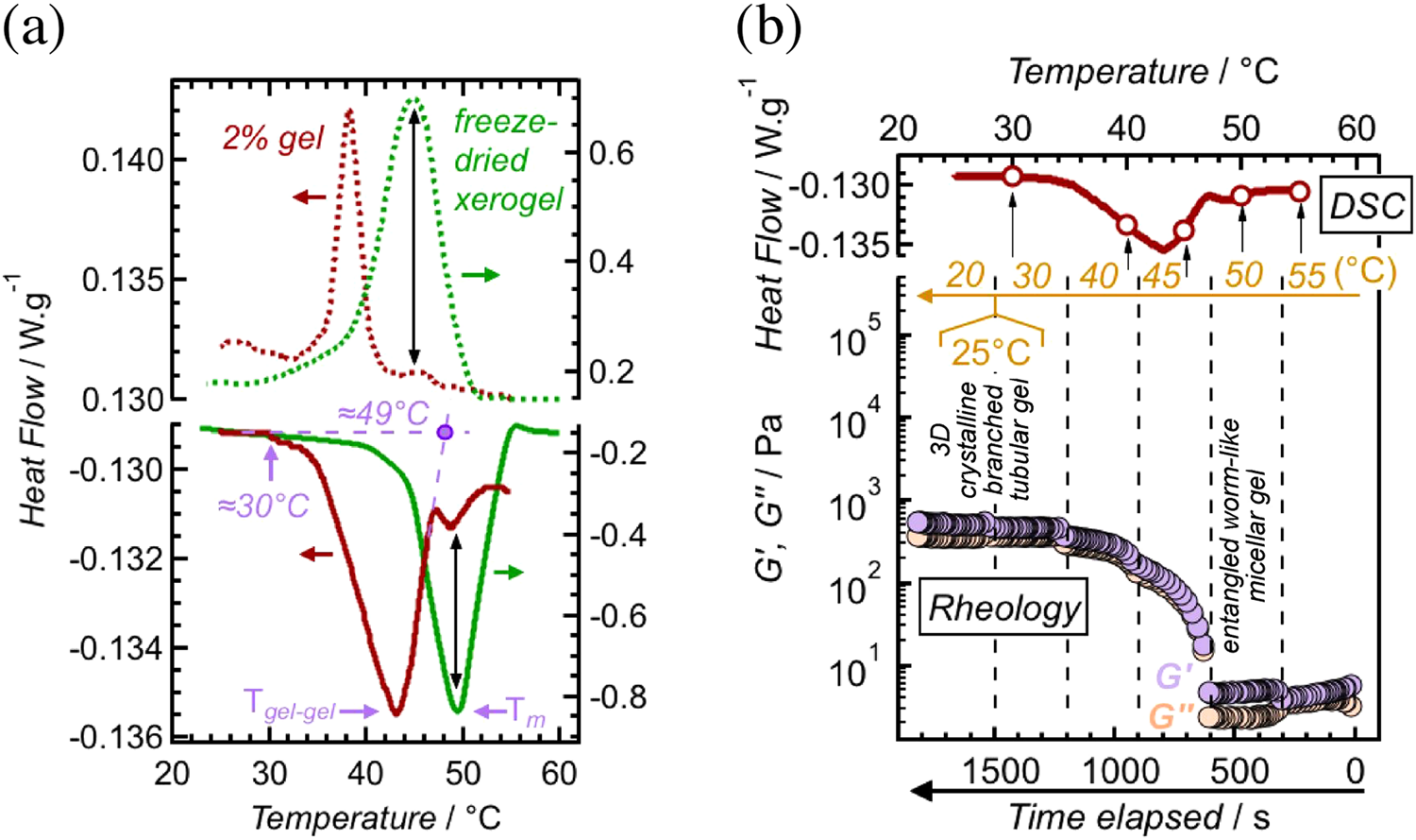
DSC and rheology data of 2% pH = 5.37 system. (**a**) DSC of the hydrogel and the corresponding freeze-dried xerogel (cooling/heating rate = 2 °C/min). The starting-point (≅ 30 °C) and the estimated end-point (≅49 °C) of the *gel-to-gel* transition were marked. (**b**) The combined DSC data (taken from Fig. 3a) and dynamic rheological data (strain = 2%; ω = 10 rad/s) of the hydrogel, where the temperature of the sample was lowered from 55 °C to 20 °C in a stepwise fashion (the waiting time between temperature changes was 300 s).

A 2% pH = 5.37 hydrogel was turbid at 25 °C and translucent at 50 °C (Fig. 1b). Reversible changes between turbidity and translucency were manifested in the light transmittance of the system during successive cooling-heating cycles (Fig. 1b). The storage modulus G′, loss modulus G′′ and complex viscosity *η* ^*^ of the hydrogel were dropped 2 orders-of-magnitude once its temperature was raised from 25 °C to 50 °C (Fig. 2a). This large change in the mechanical properties of the hydrogel was reversible and was observed during successive cooling-heating cycles (Fig. 1c). Examination of the DSC thermogram confirmed the presence of a *gel-to-gel* temperature-induced transition at 43.3 °C (Fig. 3a). The additional endothermic peak at 48.8 °C – together with 49.5 °C peak of the xerogel – were attributed to the melting of the alkyl chains (T_*m*_). The gel character of the sample was maintained (G′ > G′′) all the way from 55 °C (>T_*m*_) to 20 °C when it was cooled in a stepwise fashion (Fig. 3b).

**Figure 4:**
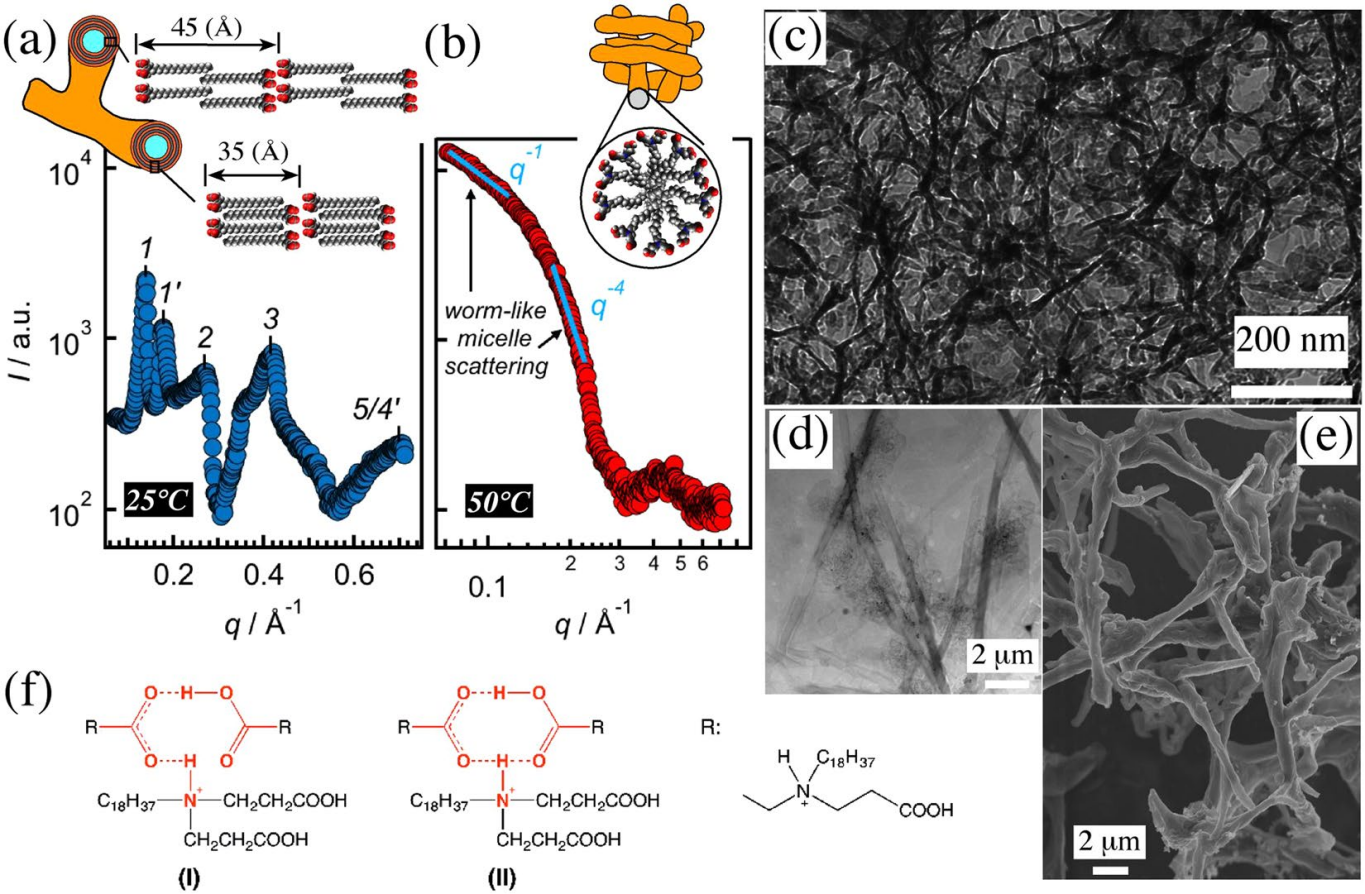
For 2% pH = 5.37 hydrogel, L-XRD, SEM, TEM and cryo-TEM data, and the results of the interpretation of IR data (IR data itself can be found in Supplementary Fig. S9). L-XRD data and the schematics of the essential features of gel microstructure at (**a**) 25 °C (crystalline bilayers) and (**b**) 50 °C (worm-like micelles), (**c**) cryo-TEM image of a 50 °C sample, (**d**) TEM image of a 25 °C sample, (**e**) SEM image of a 25 °C sample, and (**f**) possible configurations of carboxylic acid:amine 2:1 complex observed by IR at 25 °C. In (**a**) the order of observed peaks for two lamellar structures are indicated. In (**b**), the very broad peak of the L-XRD intensity pattern is centred at 0.433 (Å^−1^) [see text]. Also, the results of fits of power law functions of *I* = *Kq* ^*α*^ (*α* = −1 and −4) to the intensity pattern of (**b**) are shown (see text).

The mesoscopic structure of the hydrogel and the status of supramolecular packing of C18ADPA molecules were unravelled bellow (at 25 °C) and above (at 50 °C) the *gel-to-gel* transition temperature (T_*gel-gel*_ = 43 °C) using SEM, TEM, cryo-TEM and XRD analysis. For 25 °C hydrogel, SEM showed a 3D branched network (Fig. 4e). TEM of the 25 °C hydrogel revealed the presence of tubes (Fig. 4d). Thus, at 25 °C the hydrogel structure is made up of tubes that made branches from their cross-sections (scheme of Fig. 4a). Low-angle XRD (L-XRD) revealed that the walls of the tubes were made up of lamellae composed of polar and nonpolar layers, which were either crystalline [*d*_I_ = 45 (Å)] or exhibited fully-interdigitated alkyl-chains [*d*_II_ = 35 (Å)] (Fig. 4a; for details of structural analysis see 2.1. of Supplementary Information). For 50 °C hydrogel, on the other hand, cryo-TEM (Fig. 4c) and TEM (Supplementary Fig. S10) showed that the system contained worm-like micelles (long flexible objects^19^). This was confirmed by L-XRD data featuring typical worm-like micelle scattering in its small-angle region^20^ (Fig. 4b). This means that the data showed *q*-dependent power law intensity decreases of the forms *I*~*q* ^−1^ and *I*~*q* ^−4^ related to locally cylindrical structures and a sharp interface (Porod law), respectively. It should be pointed out that the residual of the lamellar phases could be found at 50 °C. This was corroborated by, first, the translucent nature of the sample at 50 °C (Fig. 1b), second, the existence of a very broad intensity peak in the L-XRD data (Fig. 4b), and third, the presence of tubes in the TEM image (marked by white arrows in Supplementary Fig. S10). Thus, putting side-by-side all the information obtained so far, we concluded that the observed *gel-to-gel* transition was in fact a bilayer-to-micelle phase transition.

For entangled surfactant worm-like micelles, normally two typical scenarios for the rheological behavior of the microstructure could be envisaged^21^. In one scenario^22^, micelles break and recombine rapidly in a time-scale much smaller than the overall relaxation time of the structure, resulting in a *transient* network. This means that the system has a chance to relax the applied stress through breaking of the micelles. In angular frequency (ω) sweep tests, this behavior is normally manifested as a continuously increasing G′ and a G′′ that goes through a maximum (Maxwell fluid). In the other scenario^21^, conversely, the micelle breaking time is much larger than the overall structural relaxation time, and the network is *temporally persistent*. In frequency sweep rheological tests of such materials G′ and G′′ are independent of^23, 24^ or weakly dependent on^25–27^ ω. It is this latter scenario that is observed for the frequency sweep rheological tests of our 50 °C pH = 5.37 and 5.35 samples (Fig. 2). Additionally, a typical rheological difference between entangled reverse worm-like micellar and crystalline gels^17^ was detected in strain sweep rheological measurements, where it was found that moduli plateaus at 50 °C extended to strain values 1 order-of-magnitude *more* than those at 25 °C (Supplementary Fig. S1). Thus, we concluded that the hydrogel at 50 °C consisted of an entangled temporally persistent network formed as a result of interactions due to topological constrains^21^.

And finally, what was the cause of the transition? For answering this question, we employed IR analysis for the freeze-dried samples of 25 °C and 50 °C hydrogels. The data indicated that the molecular interactions in play below and above T_*gel-gel*_ (43 °C) were different (Supplementary Fig. S9). More specifically, while for both 25 °C and 50 °C samples the presence of sodium carboxylates COO^−^Na^+^ and carboxylic acid dimers (COOH)_2_ could be verified, for the 25 °C sample a third type of molecular interaction was also identified, which involved hydrogen bonding due to proton transfer from a carboxylic acid group to a tertiary amine, resulting in a carboxylic acid:amine 2:1 complex with two possible configurations^28, 29^ (Fig. 4f). At 50 °C, the 2:1 complex disappeared, because increasing the temperature weakened hydrogen bonding. Compactness of the arrangement of three headgroups within a 2:1 complex suggested a *smaller* headgroup effective area at 25 °C compared to 50 °C. This drove the system into adopting a higher curvature self-assembly when the temperature was raised, meaning that 25 °C bilayers were changed into 50 °C worm-like micelles.

In summary, a small-molecule hydrogel based on the zwitterionic dendritic surfactant C18ADPA exhibited an unprecedented temperature-induced *gel-to-gel* phase transition (T_*gel-gel*_ = 43 °C) below the melting point of its alkyl chains (T_*m*_ = 49 °C) that involved a reversible essential transformation in the self-assembly of the system from low-temperature surfactant bilayers to high-temperature entangled surfactant worm-like micelles. C18ADPA is a new member of a recently explored family of dendritic surfactants^30–32^, and is synthesized via a simple procedure from cheap starting materials (see Methods and Materials). Such compounds have already found their way in preparation of a host of different gold^18, 31, 33, 34^, silver^35^ and platinum^36^ nanomaterials. We believe due to the added versatility brought about by this transition, new previously unexplored pathways will be opened in applications of dendritic surfactants.

## Methods and Materials

### Materials

Methylacrylate (99% + ) and octadecylamine (96% + ) were purchased from Adamas-Beta (China). Other used chemicals include methanol (99.5%, Tianjin), hexane (98%, Tianjin), sodium hydroxide (96%, Tianjin), and hydrochloric acid (36.5%, Tianjin). Distilled organic solvents and doubly distilled water were used.

### Synthesis of C18ADPA

#### Synthesis procedure of C18ADPA

The details of the synthesis procedure are as follows. Octadecylamine (8 g, 29.7 mmol) dispersed in 20 ml of methanol was added drop-wise to methylacrylate (11 ml, 120 mmol) and was left to react for 36 h. The solvent was removed under reduced pressure at 40 °C using a rotary evaporator and the colorless oil resultant was collected. The residue was dissolved in chloroform and was washed twice with aqueous 0.1 mol L^−1^ NaOH solution. To the residue, 50 ml of 1 mol L^−1^ NaOH solution was added and it was left to react at 80 °C for 10 h. In the next step, using HCl the pH was adjusted to pH = 5, the solution was filtered giving a white solid that was dried at vacuum, and was washed at least three times with hexane and methanol (yield: 60%). 1 H NMR, FT-IR, and elemental analysis were carried out using VARIAN 400 W (VARIAN medical systems, USA), VERTEX70-RAMII (Bruker, Germany), and Flash EA 1112 (Thermo Electron SPA, USA) instruments, respectively.

#### 1H NMR of C18ADPA

1 H NMR (CDCl3, ppm): δ 0.88 (t, 3 H, CH_3_), 1.25 (br, 30 H, CH_2_), 1.76 (t, 2 H,CH_2_C**H**_**2**_CH_2_N), 2.85 (t, 4 H, CH_2_C**H**_**2**_CO), 3.12 (t, 2 H,CH_2_CH_2_C**H**_**2**_N), 3.43 (t, 4H, NC**H**_**2**_CH_2_CO), 9.5 (2H, COOH). IR(KBr pellet), (cm^−1^): 3431(w), 2920(vs), 2852(vs), 1697(s), 1472(s)° MS-ESI (m/z): calculated, 413.4; found, 412.4 (M-1). Calculated for C24H47NO4: C, 69.69; H, 11.45; N, 3.39; found: C, 69.79; H, 11.51; N, 3.32

#### Sample preparation

Samples were prepared by mixing 0.1 g C18ADPA, 0.02 g NaOH and 5 ml H_2_O followed by heating up to 60 °C, so that complete dissolution was achieved. The value of pH was adjusted using 1 M HCl to pH < 5.5, and the sample was kept at 50 °C water for 2 h to form gel. Then it was left to cool down to room temperature and stayed for 12 h to equilibrate. The gel status of the solutions was examined by employing tube inversion test.

### Instrumentation

#### Determination of pH

The value of pH of the gel was adjusted using 1 M HCl at 60 °C, and then the sample was left at standstill for no less than 30 min at 25 °C. The value of pH was determined using a PHS-3C (Shanghai sheng ci instrument Co., Ltd., China) acidity meter, where the electrode was calibrated with standard buffer solutions before being used.

#### Differential Scanning Calorimetry (DSC) measurements

The DSC measurements were performed using a TA-DSCQ2000 (TA instrument GmbH, Newark) instrument. An amount of 20–30 mg of gel samples were sealed into aluminum pans, and the DSC thermograms were recorded in a temperature range of 15–65 °C (heating rate = 2 °C min^−1^) under N_2_ atmosphere. To check reproducibility, three scans of each sample were recorded. For the xerogel, 20–30 mg of the material were sealed into aluminum pans, and the DSC thermograms were recorded in a temperature range of 15–150 °C (heating rate = 2 °C min^−1^) under N_2_ atmosphere. To check reproducibility, three scans of each sample were recorded.

#### Rheology

The rheological measurements were carried out using a TA DHR-1 rheometer (TA instrument GmbH, Newark) instrument. A plate-plate geometry of 40 mm diameter, and a default gap of 1 mm were used. Frequency sweep measurements were carried out from 0.5 to 100 rad s^−1^ in the linear viscoelastic region determined via dynamic strain sweep measurements. The sample was left to equilibrate for 30 min at 50 °C and 25 °C. The rheological measurement of Fig. 3b was carried out using an angular frequency of 10 rad s^−1^ and a strain of 2%.

#### Transmission Electron Microscopy (TEM)

TEM images were recorded with a Hitachi-600 electron microscope at a working voltage of 100 kV. For the images of S11a and S11b, 20 microliters of the sample solution were spread on a copper grid coated with a carbon Formvar. After 5 min, the excess solution was wiped away with filter paper. The samples were stained with Phosphotungstic acid sodium salt (2%; pH = 6.5). The excess liquid was also wiped with filter paper after 5 min. The samples were dried at room temperature overnight.

For TEM images of S12c and S12d, a drop of sample was dispersed in 2 ml water and a carbon Formvar-coated copper grid (200 mesh) was laid on one drop of the sample solution for 10 min. Then excess solution was wiped away with filter paper.

#### Cryo-Transmission Electron Microscopy (cryo-TEM)

Samples for cryo-TEM were prepared using a Leica EM GP grid plunge device. The hydrogel samples were placed on lacy carbon grids. The grids were manually blotted with filter paper and plunged into liquid propane with a temperature close to that of the liquid nitrogen. Afterwards, the grids were stored in liquid nitrogen. The temperature was monitored and was kept constant in the chamber during all the sample preparation steps. A specimen was inserted into a cryo-transfer holder (Gatan). Cryo-TEM images were acquired on a JEOL JEM–2011 instrument.

#### Scanning Electron Microscopy (SEM)

The gels were rapidly frozen with liquid nitrogen and were allowed to freeze-dry for 24 h, which was carried out using a FD-1C-50 Freeze Dryer (Beijing Boyikang Laboratory Instruments Co., Ltd, Beijing China). The samples were then coated with gold vapor and analyzed on a Hitachi SU8010 Electron Microscopy operating at 10 kV.

#### X-ray Powder Diffraction (XRD)

XRD measurements on all of the 2% gels were made with a Bruker D8 advance diffractometer, where the source of x-ray was Cu KR radiation (wavelength = 0.15406 nm) with a voltage and current of 40 kV and 40 mA, respectively. Samples were scanned for diffraction angle ranges of 1°–10° and 10°–80°.

#### Infrared spectroscopy (IR)

The gels were rapidly frozen with liquid nitrogen and were allowed to freeze-dry for 24 h using a FD-1C-50 Freeze Dryer (Beijing Boyikang Laboratory Instruments Co., Ltd., Beijing China). IR spectra of xerogel were recorded on a VERTEX 70 Bruker spectrophotometer with KBr pellets in the 400–4000 cm^−1^ region.

## Electronic supplementary material

Supporting Information
