# Supplementary material for: A unique thermo-induced gel-to-gel transition in a pH-sensitive small-molecule hydrogel
Source: Sci Rep. 2017 Aug 16;7:8459. doi: 10.1038/s41598-017-09304-z (PMC5559595; doi:10.1038/s41598-017-09304-z)
Supplement: Supplementary file 1 — Supporting Information [file 41598_2017_9304_MOESM1_ESM.pdf]

Supplementary Information

A unique thermo-induced gel-to-gel transition in a pH-sensitive small-molecule hydrogel

Hongtao Xie,[1] Mehran Asad Ayoubi,[2] Wensheng Lu,[3] Jide Wang, [1]* Jianbin Huang[4]* and Wei Wang†[1]*

[1] Ministry Key Laboratory of Oil and Gas Fine Chemical, College of Chemistry and Chemical Engineering, Xinjiang University, Urumqi 830046, China. E-mails: awangjd@sina.cn(J.W.), wei.wang@uib.no (W.W.)
[2] Novel Drug Delivery Systems Department, Iran Polymer and Petrochemical Institute, P.O. Box 14975/112, Tehran, Iran
[3] Beijing National Laboratory for Molecular Sciences, Key Laboratory of Colloid, Interface and Chemical Thermodynamics, Institute of Chemistry, Chinese Academy of Sciences, Beijing 100190, China
[4] Beijing National Laboratory for Molecular Sciences (BNLMS), State Key Laboratory for Structural Chemistry of Unstable and Stable Species, College of Chemistry and Molecular Engineering, Peking University, Beijing 100871, China. E-mail: jbhuang@pku.edu.cn (J.H.)
† Current address of W.W. is at Department of Chemistry and Center for Pharmacy, University of Bergen, Bergen N-5007, Norway.

Table of contents

1. Rheology data
    1.1. Strain sweep tests of 2% pH=5.37 hydrogel
    1.2. Frequency sweep tests of pH=5.37 hydrogels at different concentrations
    1.3. Frequency sweep tests of 2% hydrogels at different pH values
    1.4. pH-dependent complex viscosity (η^*) for 2% hydrogels (ω=5 rad/s)
2. XRD data and analysis
    2.1. Details of analysis of XRD data of 2% pH=5.37 hydrogel
    2.2. L-XRD data of 2% pH=5.37 xerogel
    2.3. H-XRD data of 2% pH=5.37 hydrogel
    2.4. L/H-XRD data of 2% pH=4.88 hydrogel
    2.5. L/H-XRD data of 2% pH=6.02 hydrogel
3. IR data of xerogels of 2% pH=5.37 hydrogels
4. TEM data of 2% pH=5.37 hydrogel at 50°C
5. References

1. Rheology data

1.1. Strain sweep tests of 2% pH=5.37 hydrogel

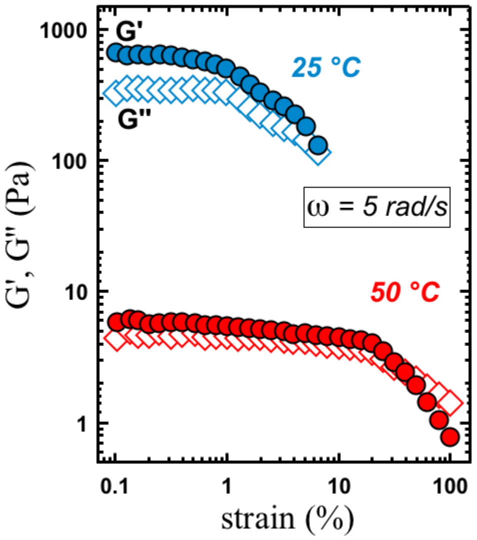

Supplementary Figure S1. Strain sweep dynamic rheological data of 2% pH=5.37 hydrogel (angular frequency of ω=5 rad/s). Filled symbols are storage modulus and hollow symbols are loss modulus.

1.2 Frequency sweep tests of pH=5.37 hydrogels at different concentrations

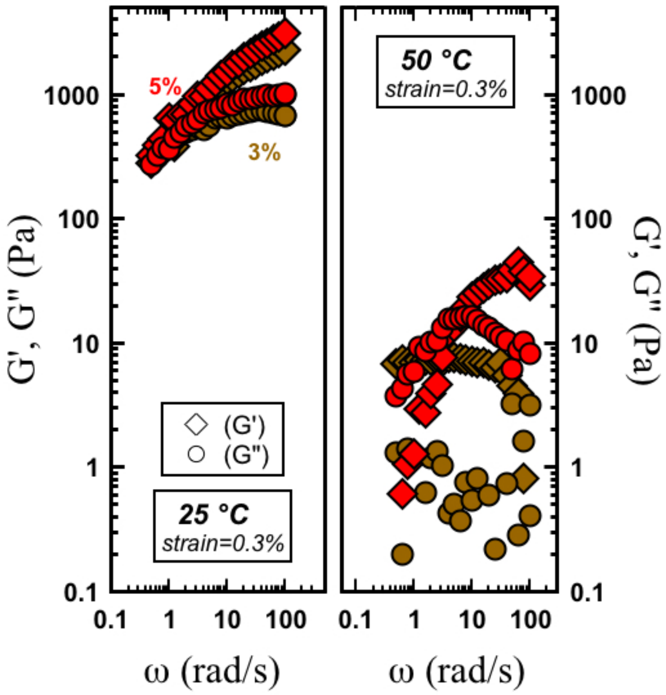

Supplementary Figure S2. Frequency sweep dynamic rheological data of 3 and 5% pH=5.37 hydrogels (strain=0.3%). Diamond symbols are storage modulus and circle symbols are loss modulus.

1.3. Frequency sweep tests of 2% hydrogels at different pH values

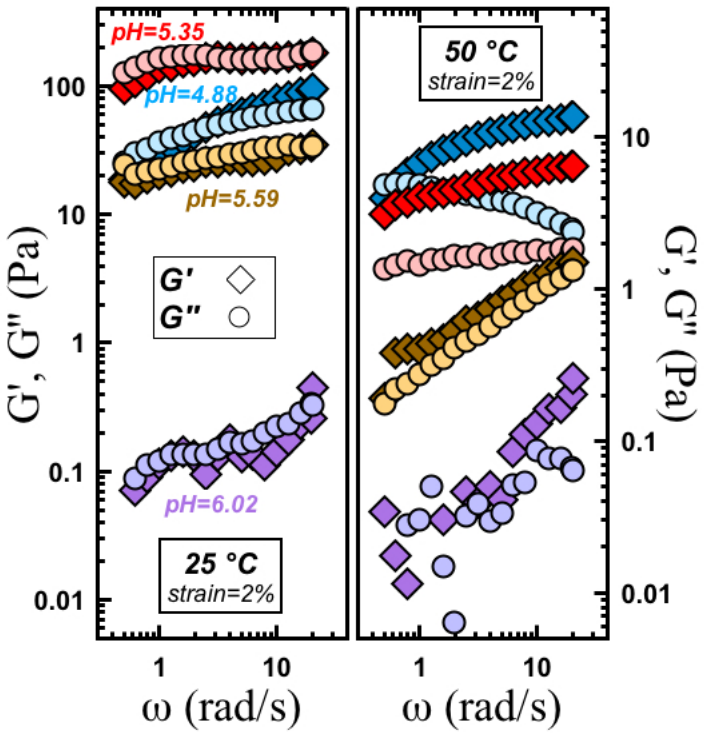

Supplementary Figure S3. Frequency sweep dynamic rheological data of 2% pH=5.35, 5.59, and 6.02 hydrogels (strain=2%). Diamond symbols are storage modulus and circle symbols are loss modulus.

1.4. pH-dependent complex viscosity (η*) for 2% hydrogels (ω=5 rad/s)

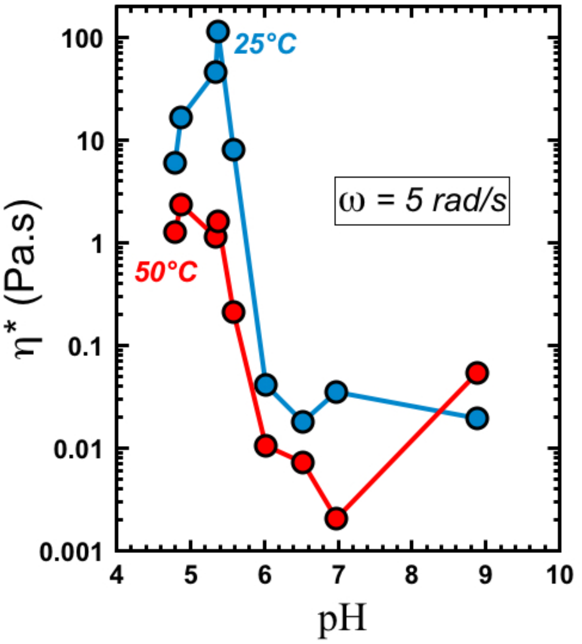

Supplementary Figure S4. pH-dependent complex viscosity (η*) for 2% hydrogels (ω=5 rad/s)

2. XRD data

2.1. Details of analysis of XRD data of 2% pH=5.37 hydrogel

For 2% pH=5.37 hydrogel at 25°C, low-angle XRD (L-XRD; Fig. 4a of the paper) showed existence of two lamellar structures (I and II). The *d*-spacing of lamellae-I is d_I=45 (Å), which was the same as the ones observed in (00*l*) reflections of crystalline lamellar bilayers of stearic acid salts[1] (C17COOX; X=Li, K), and octadecyl ammonium (C18NH3) crystals.[2] Also, there were peaks in the high-angle XRD (H-XRD) data of the sample (Fig. S6 of Supplementary). Thus, in close connection to the model of ref.[1] for salts of C17COOH, lamellae-I consisted of crystalline lamellar bilayers composed of polar and nonpolar layers (Fig. 4a of the paper). As for lamellae-II of 25°C hydrogel, a peak observed at a position very close to that of the first peak of the lamellar structure of the freeze-dried xerogel [Fig. S5 of Supplementary; *d*=36 (Å)] was indicative of the existence of a lamellar structure [d_{II}=35 (Å)], in which, alkyl chains were fully interdigitated (Fig. 4a of the paper).

2.2. L-XRD data of 2% pH=5.37 xerogel

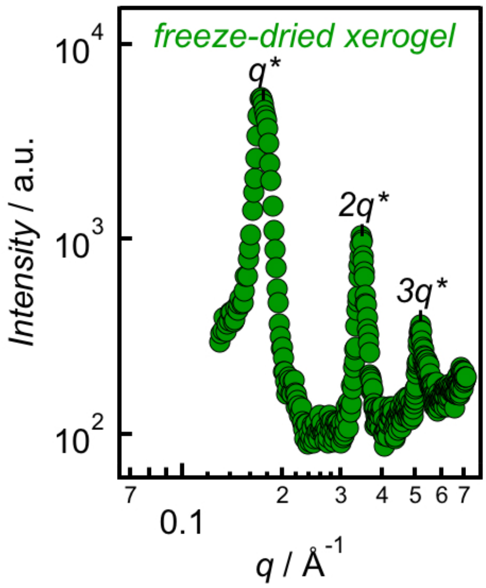

Supplementary Figure S5. L-XRD data of 2% pH=5.37 xerogel.

2.3. H-XRD data of 2% pH=5.37 hydrogel

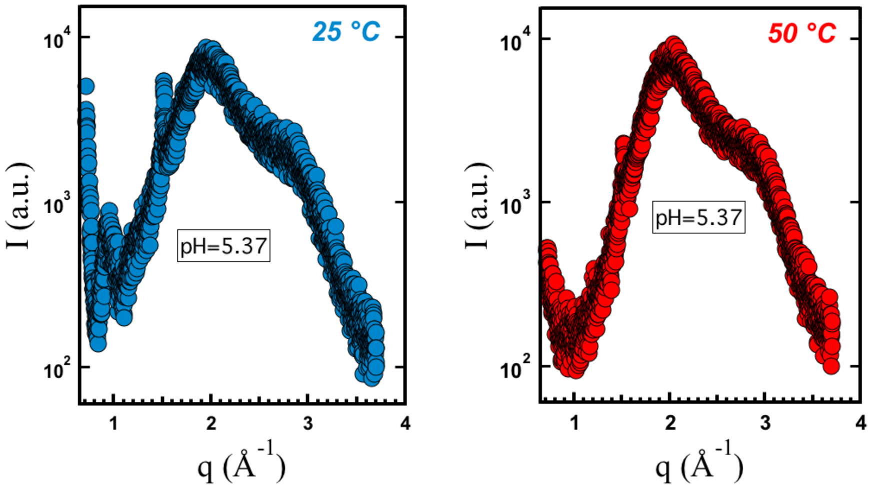

Supplementary Figure S6. H-XRD data of 2% pH=5.37 hydrogels. Water scattering gives rise to a peak [at *ca.* 2 (Å^{-1})] and a shoulder [at *ca.* 2.8 (Å^{-1})].[3]

2.4. L/H-XRD data of 2% pH=4.88 hydrogel

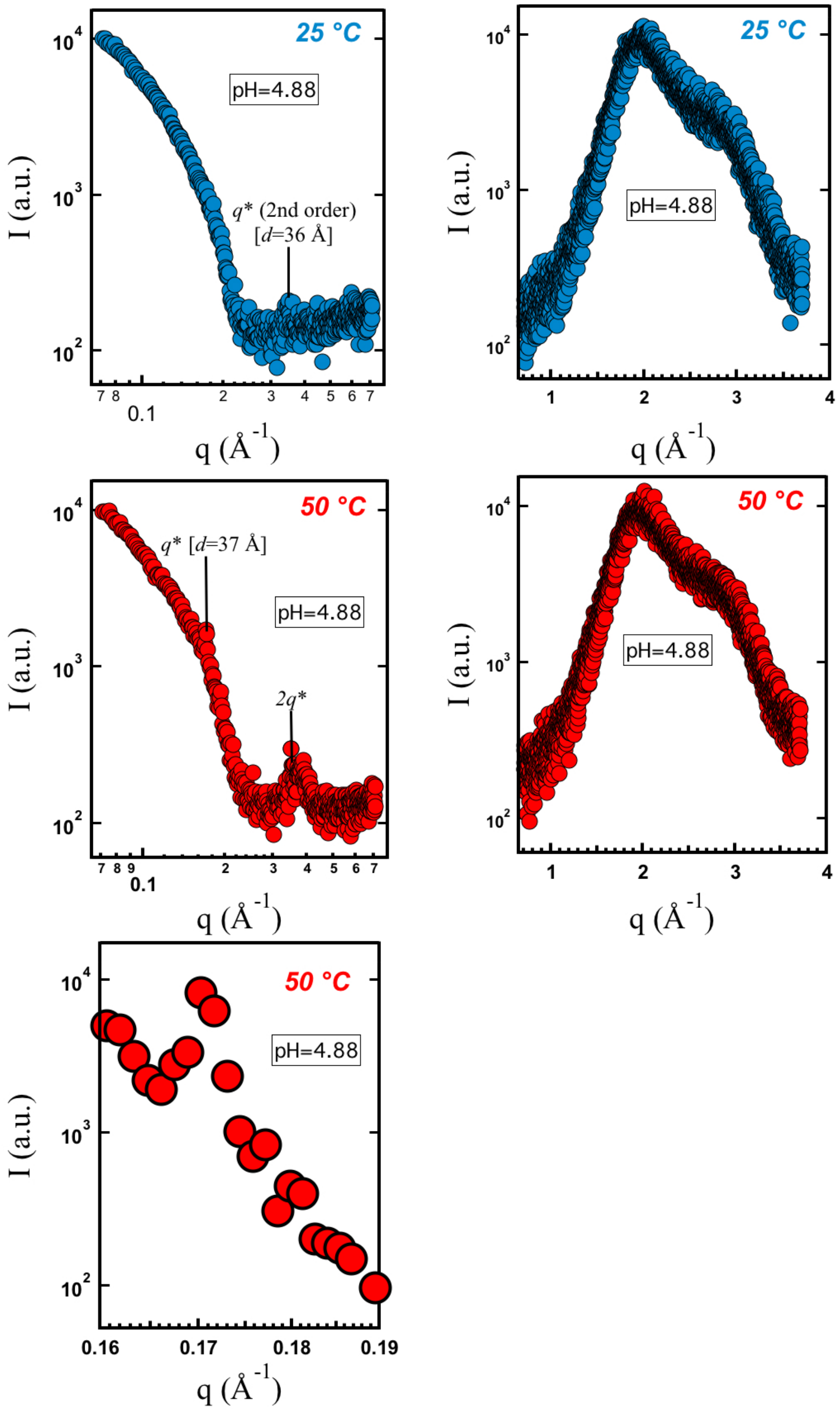

Supplementary Figure S7. XRD data of 2% pH=4.88 hydrogels. Water scattering gives rise to a peak [at *ca.* 2 (Å^{-1})] and a shoulder [at *ca.* 2.8 (Å^{-1})].[3]

<u>2.5. L/H-XRD data of 2% pH=6.02 hydrogel</u>

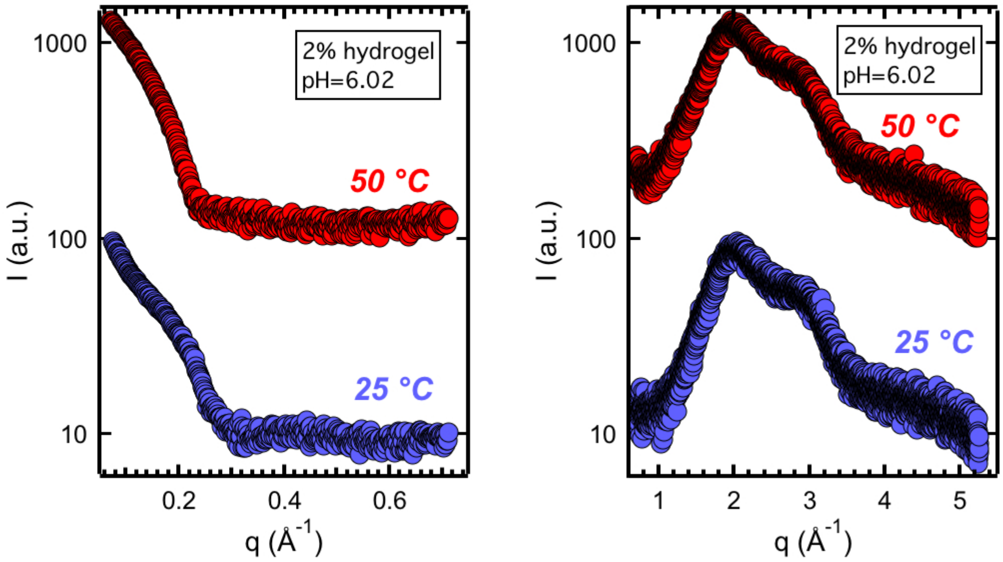

Supplementary Figure S8. XRD data of 2% pH=6.02 hydrogels. Water scattering gives rise to a peak [at *ca.* 2 (Å^{-1})] and a shoulder [at *ca.* 2.8 (Å^{-1})].[3]

3. IR data of xerogels 2% pH=5.37 hydrogels

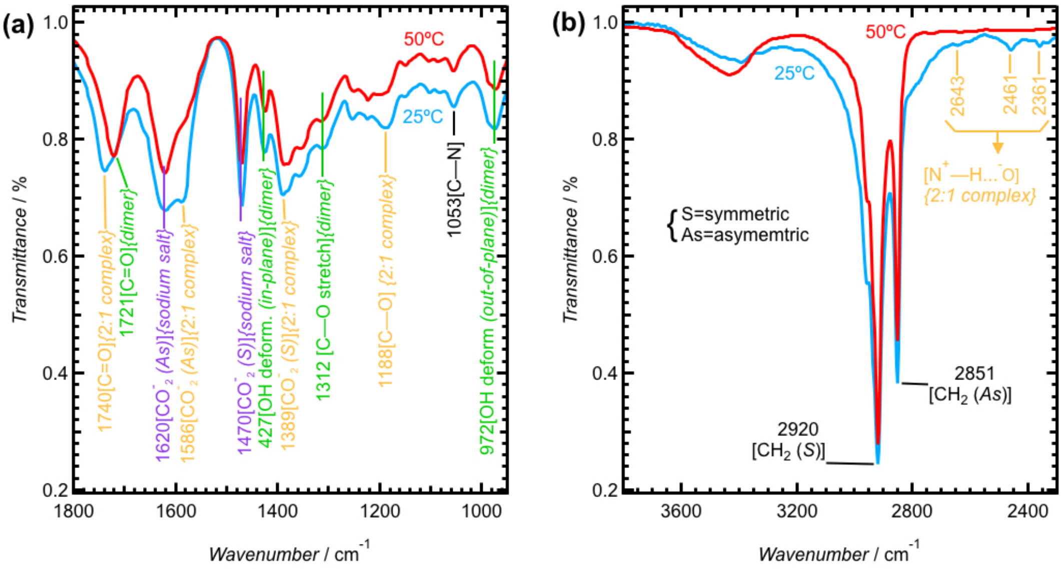

Supplementary Figure S9. IR data of 2% pH=5.37 xerogel systems.

4. TEM data 2% pH=5.37 hydrogel at 50°C

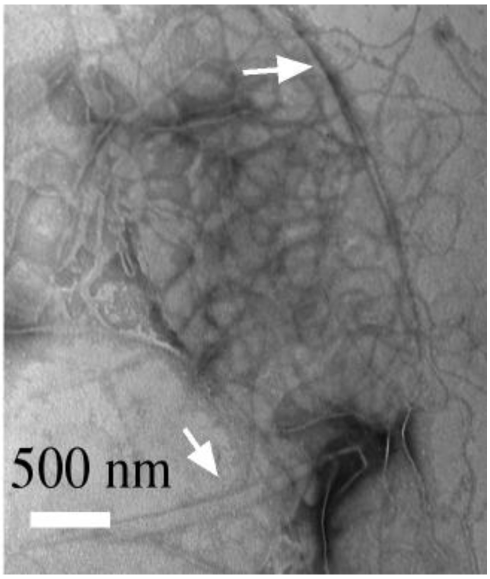

Supplementary Figure S10. TEM image of pH=5.37 hydrogel at 50°C. The arrows indicate tubes.

5. References

1 Gallot, B. & Skoulios, A. Structure des savons alcalins. *Kolloid-Z. Z. Polym.* **209**, 164-169, doi:10.1007/bf01500635 (1966).

2 Belman, N. *et al.* The Temperature-Dependent Structure of Alkylamines and Their Corresponding Alkylammonium-Alkylcarbamates. *J. Am. Chem. Soc.* **131**, 9107-9113, doi:10.1021/ja902944t (2009).

3 Huang, C. *et al.* Wide-angle X-ray diffraction and molecular dynamics study of medium-range order in ambient and hot water. *PCCP* **13**, 19997-20007, doi:10.1039/c1cp22804h (2011).
